# Sacubitril/Valsartan and Dapagliflozin in Patients with a Failing Systemic Right Ventricle: Effects on the Arrhythmic Burden

**DOI:** 10.3390/jcm13247659

**Published:** 2024-12-16

**Authors:** Giovanni Domenico Ciriello, Ippolita Altobelli, Flavia Fusco, Diego Colonna, Anna Correra, Giovanni Papaccioli, Emanuele Romeo, Giancarlo Scognamiglio, Berardo Sarubbi

**Affiliations:** Adult Congenital Heart Disease and Congenital and Familial Arrhythmias Unit, Monaldi Hospital, Leonardo Bianchi Street, 80131 Naples, Italy; gdciriello@gmail.com (G.D.C.); berardo.sarubbi@ospedalideicolli.it (B.S.)

**Keywords:** sacubitril/valsartan, dapagliflozin, systemic right ventricle, arrhythmia, supraventricular tachycardia, ventricular tachycardia

## Abstract

**Background/Objectives**: Angiotensin receptor neprilysin inhibitor (ARNI) and sodium-glucose co-transporter 2 inhibitors (SGLT2i) are essential medications in heart failure (HF) therapy, and their potential antiarrhythmic effects have been reported. Recently, ARNI and SGLT2i use for HF in adult congenital heart disease (ACHD) has been studied. However, whether any beneficial effects may be achieved on the arrhythmic burden in the complex population of ACHD with a systemic right ventricle (sRV) is still to be determined. **Methods**: We retrospectively collected all significant arrhythmic events from a cohort of patients with a failing sRV attending our tertiary care center on optimal guideline-directed medical therapy (GDMT) with ARNI and/or SGLT2i. **Results**: A total of 46 patients (mean age 38.2 ± 10.7 years, 58% male) on sacubitril/valsartan were included. Twenty-three (50%) patients were also started on dapagliflozin. After a median follow-up of 36 [Q1–Q3: 34–38] months, arrhythmic events occurred globally in 13 (28%) patients. Survival analysis showed significant reduction of clinically relevant atrial and ventricular arrhythmia at follow-up (*p* = 0.027). **Conclusions**: Our findings suggest that GDMT including sacubitril/valsartan and dapagliflozin may also offer an antiarrhythmic effect in ACHD patients with a failing sRV, by reducing the incidence of arrhythmic events at follow-up.

## 1. Introduction

Adults with congenital heart disease (ACHD) with a systemic right ventricle (sRV) have a peculiar physiology with an increased risk of heart failure (HF) [1,2], and arrhythmias throughout their lifespan and sudden cardiac death (SCD) still represent the major cause of death [3]. Advances in medical treatment and interventional and surgical techniques have significantly improved the survival of these patients, consequently increasing the proportion of patients struggling in their adult life with HF and arrhythmias. Angiotensin receptor neprilysin inhibitor (ARNI) and sodium-glucose co-transporter 2 inhibitors (SGLT2i) currently represent two pillars of HF therapy in the population with acquired heart disease. The positive effects of both ARNI and SGLT2i in heart failure with reduced ejection fraction (HFrEF) have prompted researches to investigate their potential positive effects in ACHD patients. Their use has been recently reported in ACHD patients with a failing sRV, including transposition of the great arteries (TGA) after Mustard/Senning repair or congenitally corrected TGA (ccTGA) [4,5]. Sacubitril/valsartan was demonstrated to be well tolerated and associated with favourable remodeling, improved systolic function, as well as improved clinical status, in patients with a failing sRV, supporting its use in this complex population [2]. Furthermore, dapagliflozin was associated to a favourable safety profile and promising benefits in enhancing biventricular systolic function in patients with a failing sRV [3]. Recent evidences suggest an antiarrhythmic effect of these drugs on both atrial and ventricular tachyarrhythmias in patients with HFrEF [6,7,8,9]. However, the potential antiarrhythmic benefit of ARNI and SGLT2i in the complex congenital population is still to be explored.

We aimed to assess the effects of optimal guideline-directed medical therapy (GDMT), including ARNI and SGLT2i, on the arrhythmic burden in a cohort of ACHD patients with a failing sRV.

## 2. Materials and Methods

We retrospectively reviewed the medical records of all patients with TGA after atrial switch repair or ccTGA attending our tertiary care center for ACHD between January 2017 and June 2024, focusing on those with a failing sRV, defined as a fractional area change ≤ 35% on echocardiography, which was already used as the cutoff value for our previous studies [4,5]. Among those patients, those who received GDMT for HFrEF, including sacubitril/valsartan and SGLT2i, were selected for the current study. Ethical review and approval were waived for this study due to the retrospective nature of the study. Each patient provided informed written consent.

Data at baseline and follow-up, including clinical history, medical treatment, and records from ECG Holter monitoring and implantable cardiac devices, were retrospectively collected and analysed for all enrolled patients. Follow-up visits at our unit were scheduled at 3–6-month intervals and included clinical examination, 12-lead ECG, echocardiography, and biochemistry analysis. Twenty-four-hour ECG Holter monitoring was performed periodically at 6-month intervals. Implantable device recipients were also followed with periodic in-office device interrogations (3–6-month intervals) and remote monitoring.

All significant arrhythmic episodes that occurred during an observation period of 36 months following GDMT initiation were collected from all available medical records (hospitalizations, outpatient visits, periodic ECG Holter monitoring, periodic device interrogations). For comparison, we also collected in the same patients all significant arrhythmic events from the 36 months preceding the initiation of sacubitril/valsartan therapy. Arrhythmias occurring before this observation period were excluded from our analysis. Patients who interrupted sacubitril/valsartan or dapagliflozin during the follow-up period, as well as patients who underwent catheter ablation for atrial or ventricular tachyarrhythmias, were excluded from the study.

Sustained (≥30 s) episodes of atrial arrhythmia (AA) and/or ventricular arrhythmia (VA) detected on all the available records were considered in our analysis. We also included non-sustained (<30 s) episodes of ventricular tachycardia (nsVT). As previously described elsewhere, ACHD patients with sRV who experienced life-threatening VAs or SCD had more frequent episodes of sustained AAs or nsVT prior to the event [6]. Sustained AAs and nsVT were significantly associated with an increased risk of major adverse arrhythmic events in adults with sRV [6]. Therefore, nsVT were considered in our analysis.

Statistical analysis was performed using R version 4.0.5. Continuous variables were reported as mean ± SD or median [IQR], according to data distribution. Patients were divided into two groups according to the occurrence of arrhythmic events at follow-up. Comparisons between groups were assessed with Student *t*-tests for unpaired samples or Mann–Whitney tests. Categorical variables were presented as frequencies (percentage of total). Differences in proportions were evaluated with χ^2^ tests. Survival free from clinically relevant arrhythmia (including both atrial and ventricular arrhythmias) at follow-up was compared to what was observed in the same patient population in the 36 months before initiation of GDMT. Kaplan–Meier survival curves of the historical and follow-up data were plotted with censoring the time of last follow-up or time of first events. Survival free from events was compared using log-rank tests. A *p*-value < 0.05 was considered statistically significant.

## 3. Results

From the total population of 92 patients with sRV followed at our ACHD unit, 46 (50%) patients (mean age 38.2 ± 10.7 years, 58% male) met the inclusion criteria of the present study: 30 (65%) patients with TGA after atrial switch repair and 16 (35%) patients with ccTGA. Baseline patients’ characteristics of the study cohort are summarized in Table 1. At baseline, ECG showed permanent atrial fibrillation (AF) in 3 (6%) patients. Sixteen (34%) patients were pacemaker (PM) carriers, eight (17%) patients had an implantable cardioverter-defibrillator (ICD), two (4%) an implantable loop recorder (ILR), and one (2%) a cardiac resynchronization therapy-defibrillator (CRT-D). Thirty-two (69%) had a history of arrhythmic events before study initiation. In patients with ccTGA, left atrial volume index (LAVI) was 41 [Q1–Q3: 36–63] mL/m^2^.

After appropriate washout from angiotensin-converting enzyme inhibitors (ACEi) or angiotensin receptor blockers (ARBs), all patients included were started on sacubitril/valsartan on top of their previous HF medical therapy, including beta-blockers, mineralocorticoid receptor antagonists (MRAs), and diuretics. The starting dose was 24/26 mg bid in 28 (61%) patients and 49/51 mg bid in 18 (39%) patients. Sacubitril/valsartan dosage was progressively uptitrated to the maximum tolerated dose during follow-up, as already described elsewhere [1]. The final dose of sacubitril/valsartan was 24/26 mg bid in 13 patients (28%), 49/51 mg bid in 13 patients (28%) and 97/103 mg bid in 20 patients (43%). Twenty-three (50%) patients also started dapagliflozin 10 mg daily following a mean follow-up of 17 ± 8 months from initiation of sacubitril/valsartan. Dapagliflozin was added in patients meeting the inclusion criteria as already described in our previous study, including optimal medical therapy with sacubitril/valsartan for ≥ 3 months, no previous use of SGLT2is, no univentricular physiology, systolic blood pressure > 90 mm Hg, and a glomerular filtration rate > 25 mL/min/1.73 m2 [2]. Baseline beta-blockers therapy included bisoprolol (*n* = 17, 3.4 ± 2.6 mg/daily), carvedilol (*n* = 6, 23 ± 14 mg/daily), and metoprolol (*n* = 1, 50 mg/daily). Antiarrhythmic therapy also included sotalol (*n* = 3, 80 mg/daily), amiodarone (*n* = 4, 200 mg/daily), and flecainide (*n* = 2, 100 mg/daily). Changes in antiarrhythmic therapy during follow-up are described in Table 2.

During the observation period, 2 PM carriers underwent subcutaneous ICD implantation (one for primary prevention, one for secondary prevention after hemodynamically not-tolerated VT), one patient underwent an upgrade procedure from PM to CRT-P, and one patient required lead extraction and epicardial PM reimplantation because of lead migration.

Analyzing the historical data of the 36 months before initiation of GDMT in our study population, 13 (28%) patients had experienced 20 episodes of sustained AA, including 11 episodes of macroreentrant atrial tachycardia (MRAT) (as intra-atrial reentrant tachycardia or atrial flutter) and nine episodes of AF; nine (19%) patients reported 12 episodes of VA, including four episodes of sustained VT and eight episodes of nsVT.

After a median follow-up of 36 [Q1–Q3: 34–38] months, arrhythmic events occurred globally in 13 (28%) patients: six (13%) patients reported eight episodes of sustained AA, including five episodes of MRAT and three episodes of AF while seven (15%) patients experienced seven episodes of VA, with one episode of sustained VT and six episodes of nsVT.

Comparing the number of AA and VA events occurring before and after GDMT, we observed a significant reduction in the total arrhythmic burden (Table 3).

**Figure 1:**
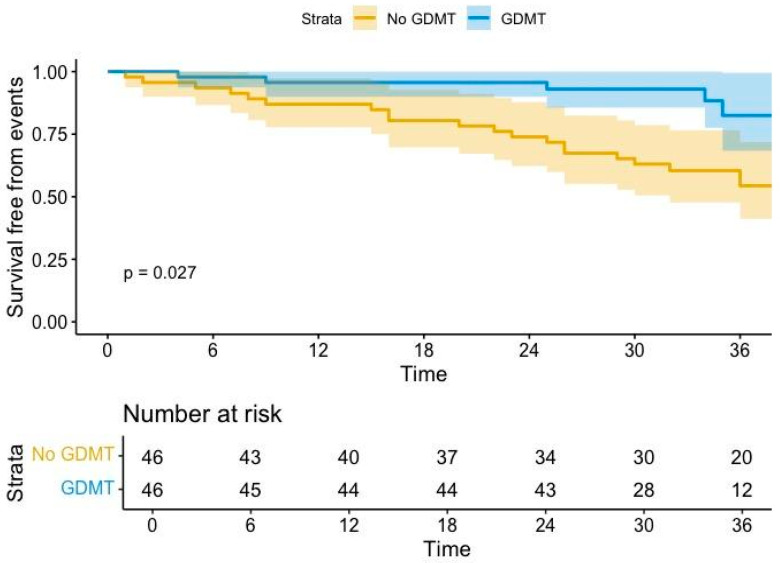
Arrhythmic burden analysis before and after GDMT (sacubitril/valsartan ± dapagliflozin). GDMT= guideline-directed medical therapy. Coloured areas indicate confidence intervals.

GDMT was associated with a reduction of arrhythmic events (HR 0.4, 95% CI, 0.17–09, *p* = 0.03). Kaplan–Meier curves confirmed this trend, depicting a significant improvement in survival free from arrhythmic events after initiation of GDMT (*p* = 0.0027, Figure 1).

Dividing patients into two groups, according to the occurrence of arrhythmic events, those with no arrhythmic events had significantly lower NT-proBNP values at the last evaluation (*p* = 0.003) (Table 4).

## 4. Discussion

Our study provides a preliminary experience supporting potential antiarrhythmic benefits from implementation of GDMT in the complex population of ACHD patients with a failing sRV over a 3-year follow-up.

Currently, ARNI and SGLT2i are key medications in HFrEF therapy in patients with acquired heart disease. Mounting data on their beneficial effects on their arrhythmic burden in HF patients, with a favourable impact on both atrial and ventricular tachyarrhythmias, have been reported [4,5].

Multiple mechanisms have been proposed to explain how sacubitril/valsartan modulates arrhythmogenesis. Sacubitril/valsartan reduces the activation of stretch-sensitive ionic channels and modulates vasoactive peptides and neurohormonal activity, increasing diuresis, vasodilation and improving wall stress and pressure/volume overload, resulting in a less excitable substrate for arrhythmias in both atrial and ventricular myocardium [6,8,9]. Consequently, it may decrease the incidence of VAs in patients with HFrEF by promoting favourable electrical remodelling, reducing ventricular hypertrophy, wall stress, inflammatory response, and myocardial fibrosis [10,11]. Sacubitril/valsartan may also impact AAs susceptibility and atrial electromechanical remodelling by inhibiting atrial fibrosis and reducing atrial volume, hypertrophy, and atrial overload [12].

Evidence on the potential antiarrhythmic effects of SGLT2i have recently emerged: a class I antiarrhythmic effect by direct inhibition of voltage-gated sodium currents was demonstrated in vitro [13]. In addition, it has been speculated that dapagliflozin may impact on cellular metabolism, leading to an improved heart energy metabolism and tissue oxygenation, reduced sympathetic overdrive, oxidative stress, and inflammatory response counteracting adverse remodelling, thus improving the substrate for both AAs and VAs [14,15]. Accordingly, SGLT2i use was associated with lower incidence of atrial flutter and/or AF in diabetic patients, as well as with reduced AA recurrences after AF ablation [15,16,17,18,19]. Moreover, both dapagliflozin and empagliflozin were demonstrated to reduce the risk of life-threatening VAs and sudden death in HFrEF patients [20,21]. Analogously, a reduced incidence of sustained VT recorded by ICD devices was found in patients with HF treated with ertugliflozin [7].

Individuals with a failing sRV are at an increased risk of arrhythmias and SCD throughout their lifespan [7,22,23,24]. Surgical incisions, suture lines, patches, baffles, surgical conduits, percutaneous devices, and areas of myocardial fibrosis due to prolonged pressure/volume overload can potentially create critical substrates for both atrial and ventricular arrhythmias, allowing conduction velocity delays, dyssynchrony, repolarization dispersion, and re-entrant circuits [22,23,24].

Medical management in patients with a failing sRV is particularly challenging and based on limited pharmacological options derived from patients with acquired heart disease [25]. Multiple studies failed to demonstrate any benefit from traditional HF medications in this peculiar group [26,27]. Moreover, despite its potential efficacy in dyssynchronous sRV, CRT device implantation is limited in this population by technical difficulties and anatomic constraints related to the underlying CHD [28,29,30].

A recent study from our group described the largest cohort of patients with a failing sRV treated with sacubitril/valsartan: this drug was well tolerated and associated with decreased NT-proBNP values, favourable remodelling, improved systolic function, as well as improved clinical status [4,31]. Analogously, dapagliflozin demonstrated a favourable safety profile and promising results in this complex population [32]. The DAPA-SERVE trial demonstrated sRV function improvement among patients in the treatment group receiving dapagliflozin [5].

This study represents the first report of the potential benefit from GDMT including sacubitril/valsartan alone or in combination with dapagliflozin on the arrhythmic burden in ACHD patients with a failing sRV. GMDT was associated with a significant reduction of clinically relevant arrhythmic events at follow-up. Interestingly, patients with no arrhythmic events at follow-up showed lower NT-proBNP values, suggesting that sacubitril/valsartan and dapagliflozin may reduce arrhythmic burden by both a direct and indirect mechanism through the reduction of both atrial and ventricular overload, the promotion of favourable reverse remodelling and, thus, potentially less arrhythmogenesis.

This study is limited by the sample size and single-center, retrospective, and non-randomized design due to the rare prevalence of the disease. Considering the limited study cohort, a comparison between patients on sacubitril/valsartan therapy alone versus dapagliflozin alone versus sacubitril/valsartan plus dapagliflozin combination was not conducted. Furthermore, not all patients in our study cohort were device carriers, so their arrhythmic burden was assessed by serial outpatient visits and periodic ECG Holter monitoring, which provides significantly different detection capabilities compared to implantable devices. Depending on periodic monitoring rather than continuous monitoring in some cases may result in underreporting of arrhythmic events, which could have underestimated the final number of arrhythmic events. We acknowledge that our preliminary data need to be confirmed with adequately powered studies with multicentric design and larger cohorts.

## 5. Conclusions

GDMT with sacubitril/valsartan and dapagliflozin may offer an antiarrhythmic benefit by reducing the burden of arrhythmic events in ACHD patients with a failing sRV. Our preliminary findings may expand upon the recently described positive effects of sacubitril/valsartan and dapagliflozin in the treatment of ACHD patients, supporting the hypothesis of a beneficial electromechanical reverse remodeling effect from adding these drugs to the medical therapy for HF in this complex population, which warrants further investigation and longer follow-up.

## Figures and Tables

**Table 1:** Characteristics of the study cohort before and after GDMT (*n* = 46).

Age (Years)	38.2 ± 10.7	
Sex	27 (58%) males	
d-TGA	30 (65%)	
ccTGA	16 (35%)	
Additional defects	11 (23%) VSD + PS	
	2 (4%) PS	
	3 (6%) VSD	
	1 (2%) MAPCAs	
PM/ICD/ILR/CRT	16 (34%)/8 (17%)/2 (4%)/1 (2%)	
	Baseline	3-year follow-up
Permanent atrial fibrillation	3 (6%)	3 (6%)
Heart rate (bpm)	66 ± 11	64 ± 12
Systolic BP (mmHg)	119 ± 12	117 ± 12
Diastolic BP (mmHg)	69 ± 11	68 ± 7
6MWT (mt)	430 [340–480]	480 [365–540]
Oxygen saturation (%)	96 ± 3.4	96.5 ± 2.1
NYHA class	I 4 pts (8%)	I 17 pts (%)
	II 39 pts (84%)	II 28 pts (%)
	III 3 pts (6%)	III 1 pts (%)
Medications for HF		
ACEi	35 pts (76%)	0
ARBs	11 pts (23%)	0
ARNI	0	46 pts (100%)
SGLT2i	0	23 pts (50%)
MRA	6 pts (13%)	8 pts (17%)
Diuretics	10 pts (21%)	6 pts (13%)

d-TGA = transposition of the great arteries, ccTGA = congenitally corrected transposition of the great arteries, VSD = ventricular septal defect, PS = pulmonary stenosis, MAPCAs = major aortopulmonary collateral arteries, PM = pacemaker, ICD = implantable cardioverter defibrillator, ILR = implantable loop recorder, CRT = cardiac resynchronization therapy, BP = blood pressure, 6MWT = six minute walking test, HF = heart failure, ARNI = Angiotensin receptor neprilysin inhibitor, SGLT2i = sodium glucose co-transporter 2 inhibitor, ACEi = angiotensin-converting enzyme inhibitor, ARB = angiotensin receptor blocker, MRA = mineralocorticoid receptor antagonist.

**Table 2:** Antiarrhythmic therapy at baseline and follow-up.

	Pre	Post
Bisoprolol	17 pts (36%) - 4(8%) 1.25 mg/daily - 7(15%) 2.5 mg/daily - 3(6%) 3.75 mg/daily - 1(2%) 5 mg/daily - 2(4%) 10 mg/daily	22 pts (47%) - 4(8%) 1.25 mg/daily - 4(8%) 2.5 mg/daily - 1(2%) 3.75 mg/daily - 1(2%) 5 mg/daily - 2(4%) 8.75 mg/daily - 1(2%) 10 mg/daily
Carvedilol	6 pts (13%) - 3(6%) 12.5 mg/daily - 2(4%) 25 mg/daily - 1(2%) 50 mg/daily	6 pts (13%) - 3(6%) 12.5 mg/daily - 2(4%) 25 mg/daily - 1(2%) 50 mg/daily
Metoprolol	1 pt (2%) 50 mg/daily	2 pts (4%) 100 mg/daily
Sotalol	3 pts (6%) - 1(2%) 40 mg/daily - 1(2%) 80 mg/daily - 1(2%) 160 mg/daily	2 pts (4%) - 1(2%) 40 mg/daily - 1(2%) 80 mg/daily
Amiodarone	4 pts (8%) 200 mg/daily	4 pts (8%) 200 mg/daily
Flecainide	2 pts (4%) 100 mg/daily	2 pts (4%) 100 mg/daily

**Table 3:** Arrhythmic burden before and after GDMT (sacubitril/valsartan ± dapagliflozin).

Arrhythmic Burden	Before GDMT 32 Episodes	After GDMT 15 Episodes
Atrial arrhythmia episodes	20 episodes - MRAT = 11 - AF = 9 In 13 patients	8 episodes - MRAT = 5 - AF = 3 In 6 patients
Ventricular arrhythmia episodes	12 episodes - Sustained VT = 4 - Non-sustained VT = 8 In 9 patients	7 episodes - Sustained VT = 1 - Non-sustained VT = 6 In 7 patients

GDMT = guideline-directed medical therapy, MRAT = macroreentrant atrial tachycardia, AF = atrial fibrillation, VT = ventricular tachycardia.

**Table 4:** Comparison between patients with and without arrhythmic events before GDMT and at last follow-up.

Before GDMT	Arrhythmic Events 20 Patients	No Arrhythmic Events 26 Patients	*p*-Value
Anatomy (d-TGA)	11 (55%)	19 (73%)	0.2
Age (years)	43.1 ± 11	32.5 ± 7	**0.04**
Sex (male)	12 (60%)	14 (53%)	0.6
NT-proBNP (pg/mL)	459 [307–955]	162 [106–273]	**0.02**
CIED recipients	9 (45%)	11 (42%)	0.8
FAC (%)	28.2 ± 4.7	29.9 ± 6.4	0.9
RV GLS (%)	−12.8 ± 2.3	−13.3 ± 3.8	0.7
TR severity - Mild - Moderate-severe	14 (70%) 6 (30%)	23 (88%) 3 (12%)	0.1
**Last follow-up**	**Arrhythmic events** **13 patients**	**No arrhythmic events** **33 patients**	***p*-value**
Anatomy (d-TGA)	9 (69%)	21 (63%)	0.7
Age (years)	36.6 ± 9	37.3 ± 10	0.8
Sex (male)	6 (46%)	21 (63%)	0.3
NT-proBNP (pg/mL)	279 [183–447]	154 [105–278]	**0.03**
CIED recipients	8 (62%)	12 (36%)	0.1
FAC (%)	33.1 ± 8.7	32.7 ± 2.1	0.9
RV GLS (%)	−14.4 ± 2.6	−15.3 ± 2.8	0.3
TR severity - Mild - Moderate-severe	9 (69%) 4 (31%)	26 (78%) 7 (22%)	0.5
History of arrhythmic events at baseline	4 (31%)	16 (48%)	0.2

d-TGA = transposition of the great arteries, NT-proBNP = N-Terminal Pro-B-Type Natriuretic Peptide, CIED = cardiac implantable electrical device, FAC = fractional area change, RV GLS = right ventricular global longitudinal strain, TR = tricuspid regurgitation.

## Data Availability

Study data are available from the corresponding author upon reasonable request.
